# Incorporating Two Crown Ether Struts into the Backbone of Robust Zirconium‐Based Metal–Organic Frameworks as Custom‐Designed Efficient Collectors for Radioactive Metal Ions

**DOI:** 10.1002/advs.202308663

**Published:** 2024-02-04

**Authors:** Lei Li, Kang Kang, Tien‐Shee Chee, Zhenjiang Tian, Qi Sun, Chengliang Xiao

**Affiliations:** ^1^ College of Chemical and Biological Engineering Zhejiang University Hangzhou 310058 P. R. China; ^2^ Department of Materials Science and Engineering Korea Advanced Institute of Science and Technology Daejeon 34141 South Korea

**Keywords:** (crown ether, cesium), metal‐organic framework, radioactive waste, strontium

## Abstract

The incorporation of crown ether into metal‐organic frameworks (MOFs) is garnered significant attention because these macrocyclic units can fine‐tune the inherent properties of the frameworks. However, the synthesis of flexible crown ethers with precise structures as the fundamental building blocks of crystalline MOFs remains a challenging endeavor, with only a limited number of transition metal examples existing to date. Herein, 18‐crown‐6 and 24‐crown‐8 struts are successfully incorporated into the skeleton of zirconium‐based MOFs to obtain two new and stable crown ether‐based MOFs, denoted as ZJU‐X100 and ZJU‐X102. These newly developed MOFs displayed high porosity and remarkable stability when exposed to various solvents, boiling water, pH values, and even concentrated HCl conditions. Thanks to their highly ordered porous structure and high‐density embedding of specific binding sites within tubular channels, these two MOFs exhibited extremely fast sorption kinetics and demonstrated outstanding performance in the uptake of strontium and cesium ions, respectively. Furthermore, the structures of Sr‐adsorbed ZJU‐X100 and Cs‐adsorbed ZJU‐X102 are solved and confirmed the precise location of Sr^2+^/Cs^+^ in the cavity of 18‐crown‐6/24‐crown‐8. This makes modular mosaic of different crown ethers into the skeleton of stable zirconium‐based MOFs possible and promote such materials have broad applications in sorption, sensing, and catalysis.

## Graphical abstract

Two new stable zirconium‐based metal–organic frameworks decorated with crown ether struts in the backbone are first fabricated. Due to the high‐density of embedding crown ether units and regular channels, ZJU‐X100 and ZJU‐X102 exhibited rapid adsorption kinetics and high adsorption capacities for Sr^2+^ and Cs^+^, respectively.

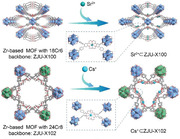

## 1 Introduction

Nuclear power is often considered as an alternative to fossil fuels, but it comes with a significant drawback: the generation of radioactive waste during the operation of nuclear power plants.^[^ ^1^ ^]^ Among them, ^90^Sr and ^137^Cs, as thermally radioactive isotopes, have strong radioactivity with long half‐lives (28.6 years and 30.17 years, respectively) and are the main sources of high‐level radioactive waste.^[^ ^2^ ^] 90^Sr is a β‐radiation emitter that releases high‐energy β‐particles. Because it is similar to Ca^2+^, once it enters the human body, it is easily combined with bones, leading to bone cancer.^[^ ^3^ ^]^^137^Cs has high mobility in the environment, posing a substantial threat to ecosystem upon its release.^[^ ^4^ ^]^^137^Cs enters the human body through the food chain, causing internal irradiation damage, and γ‐rays cause cell damage and induce the formation of tumors.^[^ ^5^ ^]^

Currently, various methods are employed to separate Sr^2+^ and Cs^+^, mainly including liquid–liquid extraction,^[^ ^6^ ^]^ precipitation,^[^ ^7^ ^]^ ion exchange,^[^ ^8^ ^]^ chromatography,^[^ ^9^ ^]^ and membrane separation.^[^ ^10^ ^]^ Among these, the adsorption method is widely used because of its simple operation, reusability, low cost, and more environmentally friendly. There have been myriad materials specifically designed and synthesized to adsorb and remove Sr^2+^ and Cs^+^, such as zeolites,^[^ ^11^ ^]^ crystalline silicotitanates (CSTs),^[^ ^12^ ^]^ layered metal sulfide materials,^[^ ^13^ ^]^ and zirconium phosphate.^[^ ^14^ ^]^ However, the separation of Sr^2+^ and Cs^+^ by purely inorganic materials is based on ion exchange and lacks specific binding sites, resulting in reduced adsorption performance, especially in the presence of high concentrations of competing cations.

**Figure 1:**
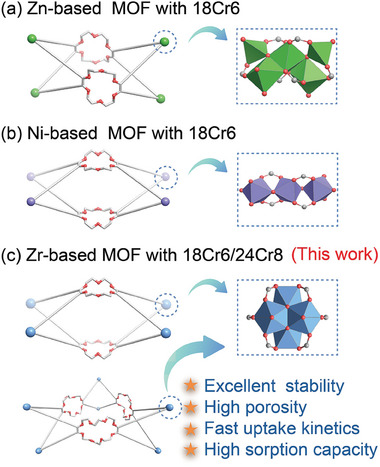
Schematic diagram of crown ether struts as the backbone of MOFs. Previous work: a) Zn‐based and b) Ni‐based MOF with 18‐crown‐6. c) Incorporating 18‐crown‐6 and 24‐crown‐8 into the backbone of Zr‐based MOFs.

Crown ethers are large cyclic molecules with ethereal oxygen atoms as repeating units.^[^ ^15^ ^]^ Their electron‐rich cavities can selectively coordinate with Sr^2+^ and Cs^+^.^[^ ^16^ ^]^ The conventional approach of integrating crown ethers into adsorbents, whether through physical or grafting methods, can block the pores and result in slow adsorption kinetics and low uptake capacity. On the other hand, metal–organic frameworks (MOFs) are crystalline porous materials constructed by repeated organic connecting units and metal nodes. It is widely applied in ion separation due to its adjustable and regular pore structure and high specific surface area.^[^ ^17^ ^]^ Several strategies have been explored to integrate crown ether units into MOFs for a range of purposes, including molecular machine,^[^ ^18^ ^]^ hydrogen storage,^[^ ^19^ ^]^ molecular recognition,^[^ ^20^ ^]^ and separation applications.^[^ ^21^ ^]^ However, incorporating crown ether struts as the backbone of MOFs to prepare the materials with high porosity and stability remains a challenge. Due to the inherent flexibility of the framework and relative fragility of nodes, it is difficult to synthesize and characterize their single‐crystal structures. To date, only two transition metal‐based MOFs with clearly resolved crown ether structures as backbones have been reported so far. Suh et al.^[^ ^19^ ^]^ were the pioneers in synthesizing such zinc‐based crown ether MOF (SNU‐200) and then Wang et al.^[21g^ ^]^ used it in the removal of radioactive Sr^2+^ with a maximum adsorption capacity of 44.37 mg g^−1^. More recently, Wang and Yuan et al.^[21f^ ^]^ replaced zinc with nickel as the metal node to fabricate MOF‐18Cr6 with 1D channel that were beneficial for Sr^2+^ transport and uptake. However, the weak coordination ability of transition metals and oxygen atoms leads to insufficient stability of these MOFs (**Figure** **1**). Zirconium metal with high‐valent states strongly interacts with carboxylate oxygen of the linkers, yielding a rich variety of MOF structures with excellent chemical stability, and high porosity.^[^ ^22^ ^]^ However, such zirconium‐based crown ether MOFs have not yet been reported. In addition, MOFs incorporated by other sizes of crown ethers in the skeleton have not been reported and the size matching effect of macrocyclic skeleton MOFs with different crown ether sizes on different cations has not been studied. In this work, we focused on embedding the modular adjustable crown ether units in the main skeleton of zirconium‐based MOFs to obtain stable adsorbents for Sr^2+^ and Cs^+^ capture and reveal the coordination mechanism of the corresponding cations in such MOFs at the atomic level. This provides an unprecedented method to customizable design of robust zirconium‐based MOF with different macrocycles skeleton, which is an unsolved challenge.

## 2 Results and Discussion

### 2.1 Synthesis and Characterization

**Figure 2:**
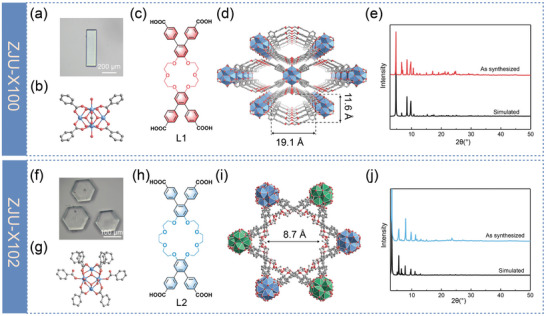
Optical image of a) ZJU‐X100 and f) ZJU‐X102 crystals. b) 8‐ connected Zr nodes of ZJU‐X100, Zr_6_O_4_(µ_3_‐OH)_4_(µ_1_‐OH)_2_(HCOO)_2_(H_2_O)_2_. c) L1 used in ZJU‐X100 (L1 = 4,4′,5,5′‐terabenzoic acid dibenzo‐18‐crown‐6). View of the 3D structures of d) ZJU‐X100 and i) ZJU‐X102. g) 8‐connected Zr nodes of ZJU‐X102, Zr_6_O_4_(µ_3_‐OH)_4_(µ_1_‐OH)(HCOO)_2_(H_2_O). PXRD patterns of e) ZJU‐X100 and j) ZJU‐X102. h) L2 used in ZJU‐X102 (L2 = 4,4′,5,5′‐terabenzoic acid dibenzo‐24‐crown‐8). Atom colors: Zr, ocean blue; O, red; C, and light grey. Hydrogen atoms were omitted for clarity.

Colorless rod‐shaped crystals (**Figure** **2**a) of [Zr_6_O_4_(µ_3_‐OH)_4_(µ_1_‐OH)_2_(HCOO)_2_(H_2_O)_2_(L1)_2_] (ZJU‐X100, L1 = 4,4′,5,5′‐terabenzoic acid dibenzo‐18‐crown‐6) were synthesized via solvent‐thermal reactions involving ZrOCl_2_·8H_2_O and H_4_L1 in N, N‐dimethylformamide (DMF) and formic acid at 120 °C for 48 h. The crystal structure was determined by single‐crystal X‐ray diffraction, which revealed that ZJU‐X100 crystallizes in the space group *Cmmm*. The framework consists of octahedral Zr_6_ nodes, each composed of six Zr atoms and four µ_3_‐OH and four µ_3_‐O groups. Additionally, two µ_1_‐OH and two water molecules, along with two formic acid molecules occupy the terminal sites of the metal cluster (Figure 2b). The coordination polymers are further assembled into a 1D diamond‐shaped channel structure along the *c*‐axis with dimensions of 11.6 × 19.1 Å (Figure 2d). The 18‐crown‐6 groups are situated on the walls of these channels. The connection between two Zr_6_ clusters and two opposite L1 ligands results in the formation of a 2D layered structure, which are further stacked in a 3D framework through hydrogen bonding and *π*–*π* interactions. Each L1 ligand acts as a (4,8)‐connected node, linking four Zr_6_ clusters via four bidentate carboxylate groups, and forming a 4,8‐connected framework with a topology of {4^20^.6^8^}{4^6^}_2_ (Figure S1, Supporting Information). According to PLATON calculations, the non‐covalent structural voids and large pores lead to a high solvent accessible volume of 55% (Figure S2, Supporting Information).

Similarly, hexagonal prism blocks (Figure 2f) of 24‐crown‐8‐based MOF [Zr_6_O_4_(µ_3_‐OH)_4_(µ_1_‐OH)(HCOO)_2_(DMF)_4_(H_2_O)(L2)_2_]·(CH_3_)_2_NH_2_ (ZJU‐X102, L2 = 4,4′,5,5′‐terabenzoic acid dibenzo‐24‐crown‐8) was synthesized under solvothermal conditions at 120 °C for 72 h employing DMF as the solvent and formic acid/trifluoroacetic acid as modulators. The metal cluster of ZJU‐X102 consists of six Zr atoms that coordinate by four µ_3_‐O and four µ_3_‐OH species, with its terminals occupied by one µ_1_‐OH, one water molecule, two formic acid molecules, and four DMF molecules (Figure 2g ; Figure S3, Supporting Information). [(CH_3_)_2_NH_2_]^+^ cations are integrated into the framework as counter ions. ZJU‐X102 is a dual‐interpenetrating framework, where the single‐layer framework represents a double‐node network. Every Zr_6_ cluster is connected to eight deprotonated L2 ligands, with two ligands coordinating in a monodentate manner and the remaining ligands in a bidentate fashion. Each L2 ligand links four Zr_6_ clusters to form 4,8‐connected framework with the Schläfli symbol of {4^14^.6^11^.8^3^}{4^3^.6^3^}_2_ (Figure S4, Supporting Information). Two identical frameworks interpenetrate each other, constructing a 3D network with channels extending along the *c*‐axis with a diameter of 8.7 Å (Figure 2i). The 24‐crown‐8 molecules are distributed on the inner walls of these channels. The network is connected by many channels and the pore vacancy is calculated to be 48% (Figure S5, Supporting Information). The phase purity of the prepared samples was confirmed by powder X‐ray diffraction analysis, showcasing an exact alignment with the simulated ones (Figure 2e,j).

**Figure 3:**
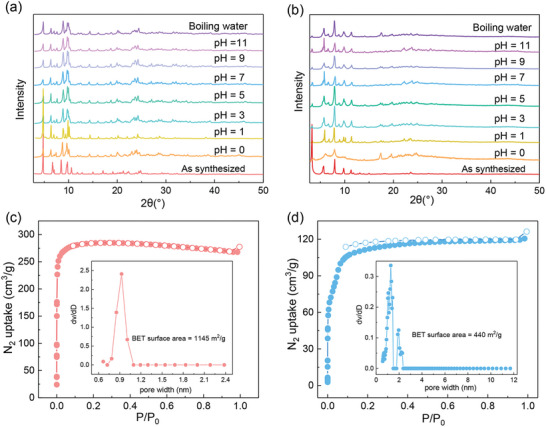
Powder X‐ray diffraction patterns of a) ZJU‐X100 and b) ZJU‐X102 after soaked in aqueous solutions in the pH range from 0 to 11 and boiling water. Nitrogen adsorption/desorption isotherms and pore‐size distribution profiles (Inset) for c) ZJU‐X100 and d) ZJU‐X102.

To further validate the chemical stability of these two materials, ZJU‐X100 and ZJU‐X102 were immersed in boiling water and aqueous solutions with pH ranging from 0 to 11 for 12 h. After exposure, both ZJU‐X100 and ZJU‐X102 retained their strong diffraction peaks (**Figure** **3**a,b), indicative of stable crystal structure. Additionally, even after immersion in 12 m hydrochloric acid solution and various organic solvents, ZJU‐X100 still maintained its crystalline structure (Figure S6, Supporting Information). The thermal stability of the materials was measured through thermogravimetric analysis. The guest molecules within the MOFs were removed below 200 °C and the decomposition of frameworks started after 350 °C (Figure S7, Supporting Information), indicative of high thermal stability of ZJU‐X100 and ZJU‐X102. Such high chemical and thermal stability might be attributed to the strong binding interaction between carboxylic acid groups and zirconium(IV). Following activation using the supercritical carbon dioxide (Sc CO_2_) method, ZJU‐X100, and ZJU‐X102 were characterized by the adsorption isotherms of nitrogen at 77 K. They both exhibited reversible type I adsorption isotherms, indicating that they held a microporous structure (Figure 3c,d). ZJU‐X100 showed a higher BET surface area of 1145 m^2^ g^−1^ while ZJU‐X102 exhibited a relatively lower BET surface area of 440 m^2^ g^−1^, possibly due to the double interpenetration characteristic of ZJU‐X102. In addition, ZJU‐X100 demonstrated a narrow pore size distribution, with an average pore width of 0.93 nm while ZJU‐X102 presented two distinct average pore widths of 1.27  and 1.96 nm.

### 2.2 Sorption Performance Studies for Sr^2+^ and Cs^+^

**Figure 4:**
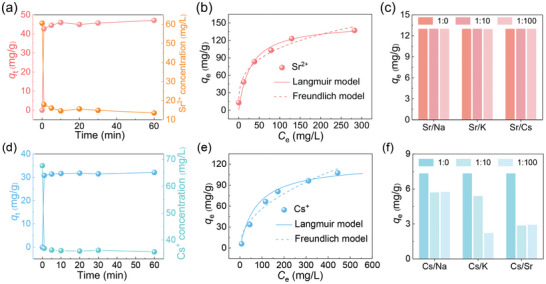
a) Adsorption kinetics, b) isotherm curves, and c) selectivity of ZJU‐X100 toward Sr^2+^. d) Adsorption kinetics, e) isotherm curves, and f) selectivity of ZJU‐X102 toward Cs^+^.

Benefiting from the crown ether skeleton and highly stable porous structure, the adsorption properties of these two MOFs for radioactive metal ions were further tested. As it is dangerous to use the ^90^Sr^2+^ and ^137^Cs^+^ compound in an unqualified laboratory, non‐radioactive surrogate Sr^2+^, and Cs^+^ was used for sorption experiments. The results indicate that these crown ether‐based MOFs exhibited good adsorption toward Sr^2+^ and Cs^+^ ions, respectively. They both reached adsorption equilibrium within 1 min (**Figure** **4**a,d), after which the concentrations of Sr^2+^ and Cs^+^ hardly changed. Such rapid adsorption kinetics compared to other sorbents (Table S1, Supporting Information) can reduce the contact time between the adsorbent and radioactive waste and improving the ability to treat leaked radioactive waste quickly, suggesting that ZJU‐X100 and ZJU‐X102 were highly suitable for the remediation of radioactive water pollution. This excellent adsorption kinetics could be attributed to the highly ordered porous structure and the high‐density embedding of crown ether units within tubular channels. Adsorption isotherms were measured with concentrations ranging from 10 to 600 ppm. Using the Langmuir model, ZJU‐X100, and ZJU‐X102 showed a maximum adsorption capacity of 149 mg g^−1^ for Sr^2+^ and 121 mg g^−1^ for Cs^+^, respectively (Figure 4b,e). Compared with previous similar adsorbents, the capacity of ZJU‐100 for Sr^2+^ were far higher than those of MOF‐18Cr6 (84.93 mg g^−1^)^[21f^ ^]^ and SNU‐200 (44.37 mg g^−1^).^[21g^ ^]^ In addition, ZJU‐X100 also showed decent selectivity for Sr^2+^ ions even when challenged by the presence of competing cation such as Na^+^, K^+^, and Cs^+^ (Figure 4c). Under the identical molar concentration of competing ions, the adsorption capacities of ZJU‐X100 for Sr^2+^ hardly decreased. Even in the presence of 100 times the concentration of Na^+^, K^+^, and Cs^+^, the adsorption capacity of ZJU‐X100 for Sr^2+^ still remained, which indicated that Sr^2+^ was well matched with the cavity of 18‐crown‐6 and attracted by the ZJU‐X100. Although ZJU‐102 showed fast adsorption and high capacity toward Cs^+^, the adsorption properties were largely affected by the competing ions (Figure 4f).

### 2.3 Sorption Mechanism Studies

**Figure 5:**
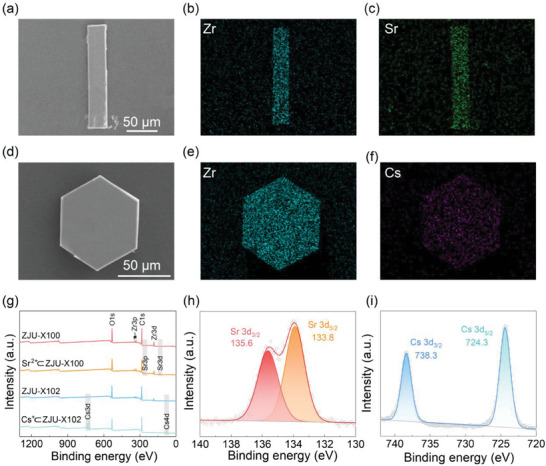
SEM imagines of a) Sr^2+^⊂ZJU‐X100 and d) Cs^+^⊂ZJU‐X102. b,c) Corresponding EDS maps of Zr (dark cyan) and Sr (green) for Sr^2+^⊂ZJU‐X100. e,f) Corresponding EDS maps of Zr (dark cyan) and Cs (purple) for Cs^+^⊂ZJU‐X102. g) XPS spectra of ZJU‐X100, Sr^2+^⊂ZJU‐X100, ZJU‐X102, and Cs^+^⊂ZJU‐X102. h) High‐resolution Sr 3d spectrum of Sr^2+^⊂ZJU‐X100. f) High‐resolution Cs 3d spectrum of Cs^+^⊂ZJU‐X102.

To explore the Sr^2+^ and Cs^+^ sorption mechanism, FT‐IR spectroscopy, SEM‐EDS, PXRD, and XPS spectra of ZJU‐X100 and ZJU‐X102 before and after sorption were analyzed. The uniform distribution of Sr and Cs in SEM‐EDS mapping images further confirmed the corresponding cation ions were successfully adsorbed onto ZJU‐X100 and ZJU‐X102 (**Figure** **5**c,f). In the FT‐IR spectra, the peaks of the two adsorbents did not change significantly before and after adsorption, indicating that ZJU‐X100 and ZJU‐X102 still maintained their chemical compositions after adsorption (Figures S8 and S9, Supporting Information). The full‐range XPS spectra of ZJU‐X100 and ZJU‐X102 before and after Sr^2+^/Cs^+^ adsorption are shown in Figure 5g. There are new peaks of the Sr 3p, Sr 3d, Cs 3d, and Cs 4d after adsorption indicates that Sr^2+^ and Cs^+^ is undoubtedly adsorbed to ZJU‐X100 and ZJU‐X102, respectively. Figure 5h,i show the high resolution XPS spectra of Sr^2+^⊂ZJU‐100 in the Sr 3d region and Cs^+^⊂ZJU‐102 in the Cs 3d region, respectively. After adsorption of Sr^2+^/Cs^+^, the two crystals exhibit characteristic peaks of corresponding cations, such as Sr 3d_3/2_ at 135.6 eV, Sr 3d_5/2_ at 133.8 eV,^[21f^ ^]^ Cs 3d_3/2_ at 738.3 eV, and Cs 3d_5/2_ at 724.3 eV.^[^ ^8^ ^]^ As shown in Figures S10a and S11a (Supporting Information), a shift of 0.2  and 0.3 eV for the C─O peak is observed in the high‐resolution O 1s XPS spectra after the adsorption experiment and new peaks attributed to O─Sr and O─Cs appeared at 531.7 eV after the adsorption of Sr^2+^ and Cs^+^ by ZJU‐X100 and ZJU‐X102, respectively. This indicated that the interaction between 18‐crown‐6/24‐crowm‐8 groups and Sr^2+^/Cs^+^.^[21f^ ^]^ A shift of 0.1 eV for the C─O peak is observed in the high‐resolution C 1s XPS spectra after the adsorption experiment (Figures S10b and S11b, Supporting Information), which further proved the combination of Sr^2+^/Cs^+^ and 18‐crown‐6/24‐crown‐8 struts. The peaks of ZJU‐X100 and ZJU‐X102 in the PXRD patterns (Figures S12 and S13, Supporting Information) all remained after adsorbed cation ions. Moreover, these two MOFs after sorption still retained the rod‐shaped and hexagonal prism crystal morphology as before (Figure 5a,d), indicating that and the skeleton of ZJU‐X100 and ZJU‐X102 remained stable after the adsorption process.

**Figure 6:**
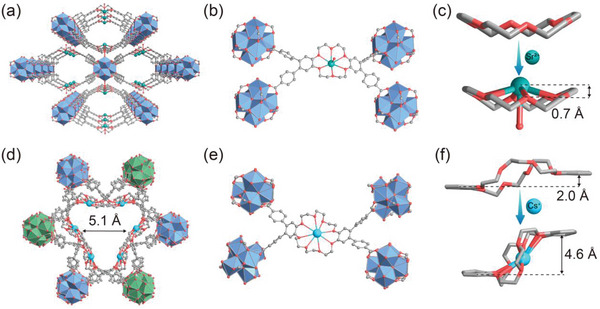
Single‐crystal X‐ray structures of a) Sr^2+^⊂ZJU‐X100 and d) Cs^+^⊂ZJU‐X102. Encapsulation of b) Sr^2+^ within the cavity of 18‐crown‐6 group of ZJU‐X100 and e) Cs^+^ within the cavity of 24‐crown‐8 group of ZJU‐X102. c) Representation of the Sr^2+^ inclusion complex in Cs^+^⊂ZJU‐X100, showing the relative positions of Sr^2+^ and crown ether and coordinated water molecules. f) Conformation of dibenzo‐24‐crown‐8 in ZJU‐X102 before and after complexation with Cs^+^. Atom colors: Sr, green; Cs, sky blue; O, red; C, and light grey. Hydrogen atoms were omitted for clarity.

To investigate the adsorption sites of cations in frameworks in detail, we first obtained the precise crystallographic information of the adsorbed crystal, Sr^2+^⊂ZJU‐X100, and Cs^+^⊂ZJU‐X102. It is found that ZJU‐X100 experienced a transformation from single crystal to single crystal (**Figure** **6**a). In the structure of Sr^2+^⊂ZJU‐X100, Sr^2+^ is trapped in the central framework of the crown ether (Figure 6b), adopting a twisted hexagonal pyramid geometry by coordinating with six oxygen atoms of the crown ether and one water molecule and forming a crown ether inclusion compound with 18‐crown‐6. Its rhombic pores expand from 11.6 Å × 19.1 Å to 12.6 Å × 19.0 Å (Figure S14, Supporting Information). The crown ether unit maintains the same boat‐shaped conformation with original ZJU‐100 and the bending angle changes slightly from 25.5° to 29°, which indicates that 18‐crown‐6 has good size matching with Sr^2+^, further explaining the excellent selectivity of ZJU‐X100 toward Sr^2+^. The distance of the Sr─O bonds ranges from 2.7 to 2.8 Å and the distance between Sr^2+^ and the oxygen atom of the water molecule is 2.5 Å (Figure S15, Supporting Information). In addition, Sr^2+^ ion is located 0.7 Å above the ring plane formed by the six oxygen atoms of the crown ether and is coordinated with a water molecule in the axial direction (Figure 6c).

Careful inspection of Cs^+^⊂ZJU‐X102 showed that Cs^+^ is located in the center of the octagon composed of polyether oxygen atoms (Figure 6e) and form a new eight‐coordination center composed of eight Cs‐O bonds with a distance range of 3.1 to 3.4 Å (Figure S16, Supporting Information). Compared with ZJU‐X102, 24‐crown‐8 in Cs^+^⊂ZJU‐X102 undergoes a conformational transition and caused the pores along the *c*‐axis direction to change from 8.7 to 5.1 Å (Figure 6d) in order to adapt to the chelation of Cs^+^. After the formation of the complex, 24‐crown‐8 changed to a more twisted chairlike structure. The distance between the planes formed by the phenyl group changed from 2.0 to 4.6 Å (Figure 6f). The conformational change requires overcoming the corresponding energy barrier, which explains the adsorption of Cs^+^ by ZJU‐X102 is more affected by other ions than ZJU‐X100. These comprehensive structural details provide insights into the sorption selectivity, binding sites, and coordination modes of zirconium‐based metal–organic frameworks with crown ether struts for Sr^2+^/Cs^+^.

## 3 Conclusion

In summary, two new crown ether incorporated in the backbone of zirconium‐based metal–organic frameworks (ZJU‐X100 and ZJU‐X102) were first synthesized via the solvothermal method. The resulting MOFs exhibited high specific surface area and outstanding stability under various conditions. Owing to the high‐density of embedding crown ether units and regular channels, ZJU‐X100, and ZJU‐X102 exhibited rapid adsorption kinetics and high adsorption capacities for Sr^2+^ and Cs^+^, respectively. Even in the presence of 100 times competitive ions, ZJU‐X100 showed excellent adsorption performance for Sr^2+^. SEM–EDS, FT‐IR, XPS spectra, and PXRD patterns proved that Cs^+^/Sr^2+^ was adsorbed into ZJU‐X100/ZJU‐X102. After adsorbing correspounding cation ions, ZJU‐X100, and ZJU‐X102 underwent a SC–SC structural transformation, in which Sr^2+^ and Cs^+^ snugly nestled within the 18‐crown‐6 and 24‐crown‐8 to form 1:1 inclusion complexes, respectively. This study contributes valuable knowledge to the field of metal–organic frameworks contained crown ether struts, promising a wealth of opportunities in a broad application across sorption, sensing, catalysis, and membrane transport.

## Conflict of Interest

The authors declare no conflict of interest.

## Supporting information

Supporting Information

## Data Availability

The data that support the findings of this study are available from the corresponding author upon reasonable request.

## References

[1] J. Li , X. Wang , G. Zhao , C. Chen , Z. Chai , A. Alsaedi , T. Hayat , X. Wang , Chem. Soc. Rev. 2018, 47, 2322. 29498381 10.1039/c7cs00543a

[2] a) N. Ding , M. G. Kanatzidis , Nat. Chem. 2010, 2, 187; 21124475 10.1038/nchem.519

[3] T. T. Lv , W. Ma , D. Zhang , T. Zhang , J. H. Tang , X. Zeng , M. L. Feng , X. Y. Huang , Chem. Eng. J. 2022, 435, 134906.

[4] S. J. Datta , W. K. Moon , D. Y. Choi , I. C. Hwang , K. B. Yoon , Angew. Chem. 2014, 126, 7331. 10.1002/anie.201402778 24853915

[5] H. Y. Sun , B. Hu , T. T. Lv , Y. L. Guo , Y. X. Yao , L. Yang , M. L. Feng , X. Y. Huang , Small 2023, 19, 2208212. 10.1002/smll.202208212 36916691

[6] J. Sharma , P. N. Khan , P. S. Dhami , P. Jagasia , V. Tessy , C. Kaushik , Sep. Purif. Technol. 2019, 229, 115502.

[7] K. Kosaka , M. Asami , N. Kobashigawa , K. Ohkubo , H. Terada , N. Kishida , M. Akiba , Water Res. 2012, 46, 4397. 22717151 10.1016/j.watres.2012.05.055

[8] Y. J. Gao , M. L. Feng , B. Zhang , Z. F. Wu , Y. Song , X. Y. Huang , J. Mater. Chem. A 2018, 6, 3967.

[9] A. Zhang , J. Li , Y. Dai , L. Xu , Sep. Purif. Technol. 2014, 127, 39.

[10] J. Wang , S. Zhuang , Rev. Environ. Sci. Bio/Technol. 2019, 18, 231.

[11] a) X. H. Fang , F. Fang , C. H. Lu , L. Zheng , Nucl. Eng. Technol. 2017, 49, 556;

[12] a) A. Clearfield , D. G. Medvedev , S. Kerlegon , T. Bosser , J. D. Burns , M. Jackson , Solvent Extr. Ion Exch. 2012, 30, 229;

[13] a) D. Sarma , C. D. Malliakas , K. Subrahmanyam , S. M. Islam , M. G. Kanatzidis , Chem. Sci. 2016, 7, 1121; 29910868 10.1039/c5sc03040d PMC5975790

[14] Z. Li , E. L. Vivas , Y. J. Suh , K. Cho , J. Environ. Chem. Eng. 2022, 10, 107333.

[15] M. Matviyishyn , A. Białońska , B. Szyszko , Angew. Chem., Int. Ed. 2022, 61, e202211671. 10.1002/anie.202211671 PMC10098552 36214485

[16] J. Wang , S. Zhuang , Nucl. Eng. Technol. 2020, 52, 328.

[17] K. Jin , B. Lee , J. Park , Coord. Chem. Rev. 2021, 427, 213473.

[18] a) Q. Li , C. H. Sue , S. Basu , A. K. Shveyd , W. Zhang , G. Barin , L. Fang , A. A. Sarjeant , J. F. Stoddart , O. M. Yaghi , Angew. Chem., Int. Ed. 2010, 49, 6751; 10.1002/anie.201003221 20715253

[19] D. W. Lim , S. A. Chyun , M. P. Suh , Angew. Chem. 2014, 126, 7953. 10.1002/anie.201404265 24939240

[20] Q. Li , W. Zhang , O. Š. Miljanić , C. H. Sue , Y. L. Zhao , L. Liu , C. B. Knobler , J. F. Stoddart , O. M. Yaghi , Science 2009, 325, 855. 19679809 10.1126/science.1175441

[21] a) L. Bai , B. Tu , Y. Qi , Q. Gao , D. Liu , Z. Liu , L. Zhao , Q. Li , Y. Zhao , Chem. Commun. 2016, 52, 3003; 10.1039/c5cc09935h 26785426

[22] Z. Chen , S. L. Hanna , L. R. Redfern , D. Alezi , T. Islamoglu , O. K. Farha , Coord. Chem. Rev. 2019, 386, 32.

