# Supplementary material for: Incorporating Two Crown Ether Struts into the Backbone of Robust Zirconium‐Based Metal–Organic Frameworks as Custom‐Designed Efficient Collectors for Radioactive Metal Ions
Source: Adv Sci (Weinh). 2024 Feb 4;11(14):2308663. doi: 10.1002/advs.202308663 (PMC11005732; doi:10.1002/advs.202308663)
Supplement: Supplementary file 1 — Supporting Information [file ADVS-11-2308663-s001.pdf]

ADVANCED SCIENCE
Open Access

Supporting Information

for *Adv. Sci.*, DOI 10.1002/advs.202308663

Incorporating Two Crown Ether Struts into the Backbone of Robust Zirconium-Based Metal–Organic Frameworks as Custom-Designed Efficient Collectors for Radioactive Metal Ions

*Lei Li, Kang Kang, Tien-Shee Chee, Zhenjiang Tian, Qi Sun and Chengliang Xiao**

Supporting Information

Incorporating Two Crown Ether Struts into the Backbone of Robust Zirconium-based Metal-Organic Frameworks as Custom-Designed Efficient Collectors for Radioactive Metal Ions

*Lei Li, Kang Kang, Tien-Shee Chee, Zhenjiang Tian, Qi Sun, and Chengliang Xiao**

Materials and instrumentation

All starting materials were purchased from commercial companies and used without further purification. Powder X-ray diffraction (PXRD) patterns were recorded on a Rigaku Ultima IV diffractometer equipped with Cu Kα radiation from 3° to 60° with a step of 0.02°. The porous properties of the samples were measured by N_2 physisorption at 77 K on an automated specific surface area and micropore analyzer (AUTOSORB-IQ2-MP). TG analysis was performed in a nitrogen atmosphere with a rate of 10 °C min^{-1} using a TA Instruments SDT Q600. X-ray photoelectron spectroscopy (XPS) spectra were acquired on a Thermo Scientific ESCALAB 250Xi. FT-IR spectra were obtained on a Nicolet 6700 spectrometer in KBr pellets in the wavelength range of 4000 – 400 cm^{-1}. EDS images were acquired on a Scanning Electron Microscopy with an Energy Dispersive X-ray Spectrometer (SEM–EDS, Hitachi SU8010). The concentrations of Sr^{2+} and Cs^{+} in solutions were analyzed by an ICP–MS (Agilent Technologies 7800). The Single-crystal X-ray diffraction data collection of ZJU-X100, $Sr^{2+}\subset$ZJU-X100 and $Cs^{+}\subset$ZJU-X102 were recorded on a Bruker D8-Venture diffractometer with a Turbo X-ray Source (Cu–Kα radiation). The Single-crystal X-ray diffraction data collection of ZJU-X102 was recorded on a Bruker D8-Venture diffractometer with an X-ray Source (Ga–Kα radiation). The data frames were collected using the program APEX 3 and processed using the program SAINT routine in APEX 3. The crystal structures were refined by the full-matrix least-squares on F^2 using the SHELXTL-2018 program.

Experimental section

Synthesis of Linkers.

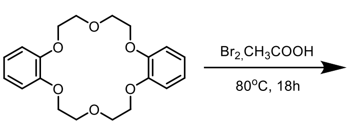

4,4’,5,5’-tetrabromodibenzo-18-crown-6. Dibenzo 18-crown-6 (12.50 g, 27.7 mmol) was dissolved in acetic acid (230 ml). Bromine (13.30 ml, 260.4 mmol) was added to the solution, and the mixture was stirred for 18 h at 80 ˚C. After cooling the solution, the orange powder was filtered and washed with MeOH and water to give white residue (19.25 g, 82.12%).^{1}H NMR (500 MHz, $CDCl_3$, 298 K): δ = 7.03 (s, 4 H), 4.10 (t, 8 H), 3.96 (t, 8 H).

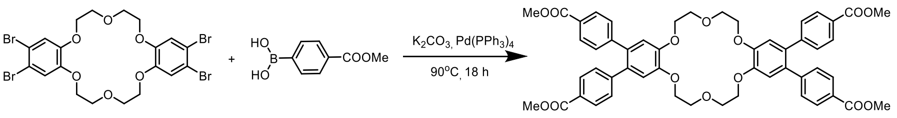

4,4',5,5'-tetrabenzoate dibenzo-18-crown-6. 4,4',5,5'-tetrabromodibenzo-18-crown-6 (11.30 g, 16.71 mmol), 4-methoxycarbonylphenylboronic acid (18.06 g, 100.35 mmol), K_2CO_3(23.1 g, 167.3 mmol), and tetrakis(triphenylphosphine) palladium (1.35 g, 1.17 mmol) were added into a 1 L Schleck flask. The flask was pumped under vacuum and refilled with Ar gas three times, and then the degassed solvent of 1,4-dioxane/CH_3OH (540 mL/180 mL) were transferred into the Schleck flask. The mixture solution was heated at 90 °C for 18 h under an Ar atmosphere. After that the reaction mixture was cooled to room temperature. It was concentrated by a rotary evaporator and washed by saturated NaCl solution. Finally, the crude product was recrystallized with ethyl acetate and CH_2Cl_2 to obtain a white solid. Yield: 9.18 g (61.24 %). ^{1}H NMR (500 MHz, $CDCl_3$, 298 K): δ = 7.87(d,8 H), 7.14 (d, 8 H), 6.94 (s, 4 H), 4.26 (t, 8 H), 4.08 (t, 8 H), 3.89 (s, 12 H).

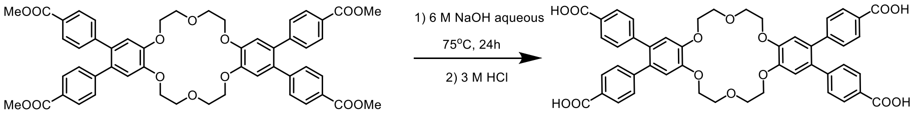

4,4’5,5’-terabenzoic acid dibenzo-18-crown-6. 4,4',5,5'-tetrabenzoate dibenzo-18-crown-6 (14.7 g, 0.966 mmol) was dissolved in 550 mL of CH_3OH, and 183 mL of 6 M NaOH aqueous solution was added. The mixture was stirred under 75 °C for 24 h. After cooling to room temperature, it was concentrated under vacuo to remove methanol. 3 M HCl was added to the above solution and adjusted to pH = 1. white precipitate was obtained by centrifugation and washed with water and dried in vacuum oven at 70 °C for 12 h. Yield: 11.58 g (84.03%). ^{1}H NMR (500 MHz, DMSO-d_6, 298 K): δ = 7.78 (d, 8 H), 7.22 (d, 8 H), 7.03 (s, 4 H), 4.22 (t, 8 H), 3.88 (t, 8 H).

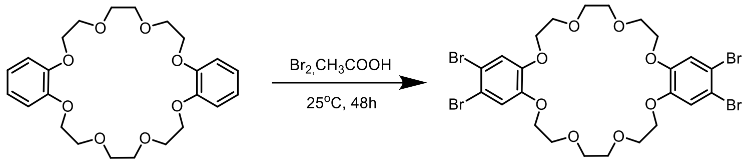

4,4’,5,5’-tetrabromodibenzo-24-crown-8. Dibenzo 24-crown-8 (9.00 g, 20.07 mmol) was dissolved in acetic acid (120 ml). Bromine (8.1 ml, 153.78 mmol) was added to the solution, and the mixture was stirred for 48 h at 25 ˚C. After cooling the solution, the orange powder was filtered and washed with MeOH and water to give a white residue (10.68 g, 69.6 %).^{1}H NMR (500 MHz, $CDCl_3$, 298 K): δ = 7.06 (s, 4 H), 4.10 (t, 8 H), 3.89 (t, 8 H), 3.79 (s, 8 H).

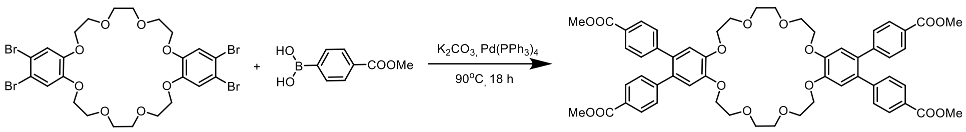

4,4',5,5'-tetrabenzoate dibenzo-24-crown-8. 4,4',5,5'-tetrabromodibenzo-24-crown-8 (11.00 g, 14.44 mmol), 4-methoxycarbonylphenylboronic acid (15.46 g, 85.9 mmol), K_2CO_3 (19.79 g, 143.14 mmol), and tetrakis(triphenylphosphine) palladium (1.15 g, 0.99 mmol) were added into a 1 L Schleck flask. The flask was pumped under vacuum and refilled with Ar gas three times, and then the degassed solvent of 1,4-dioxane/CH_3OH (540 mL/180 mL) were transferred into the Schleck flask. The mixture solution was heated at 90 °C for 18 h under an Ar atmosphere. After that the reaction mixture was cooled to room temperature. It was concentrated by a rotary evaporator and washed by saturated NaCl solution. Finally, the crude product was recrystallized with ethyl acetate and CH_2Cl_2 to obtain a white solid. Yield: 8.62 g (60.78 %). ^{1}H NMR (500 MHz, $CDCl_3$, 298 K): δ = 7.86(d,8 H), 7.14 (d, 8 H), 6.94 (s, 4 H), 4.24 (t, 8 H), 3.97 (t, 8 H), 3.89 (s, 12 H).

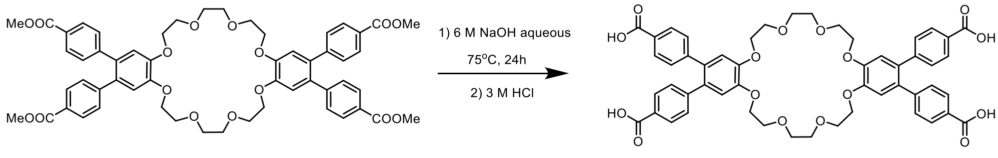

4,4’,5,5’-terabenzoic acid dibenzo-24-crown-8. 4,4',5,5'-tetrabenzoate dibenzo-24-crown-8 (13.4 g, 0.966 mmol) was dissolved in 460 mL of CH_3OH, and 153 mL of 6 M NaOH aqueous solution was added. The mixture was stirred under 75 °C for 24 h. After cooling to room temperature, it was concentrated under vacuo to remove methanol. 3 M HCl was added to the above solution and adjusted to pH=1. White precipitate was obtained by centrifugation and washed with water and dried in vacuum oven at 70 °C for 12 h. Yield: 10.68 g (84.56 %). ^{1}H

NMR (500 MHz, DMSO-d_6, 298 K): δ = 7.78 (d, 8 H), 7.21 (d, 8 H), 7.04 (s, 4 H), 4.21 (t, 8 H), 3.81 (t, 8 H), 3.71 (s, 8 H).

Synthesis of ZJU-X100. The ligand of L1(4,4',5,5'-terabenzoic acid dibenzo-18-crown-6) (10 mg, 0.0119 mmol) and $ZrOCl_2{\cdot}8H_2O$ (11.5 mg, 0.0356 mmol) were added in 6 mL of DMF/formic acid (v/v, 4/2) solution. The mixture was placed into a Teflon-lined stainless-steel autoclave and heated at 120 °C for 48 h. After cooling to room temperature, the crystals were isolated by filtration, then washed with DMF, EtOH and dried at 50 °C for 12 h (Yield: 53% based on L1). The crystal data of ZJU-X100 is presented in **Table S2**.

Synthesis of ZJU-X102. The ligand of L2(4,4',5,5'-terabenzoic acid dibenzo-24-crown-8) (45 mg, 0.0484 mmol), $ZrOCl_2{\cdot}8H_2O$ (17.5 mg, 0.0543 mmol) and 80 μL TFA were added in 6 mL of DMF/formic acid (v/v, 4/2) solution. The mixture was placed into a Teflon-lined stainless-steel autoclave and heated at 120 °C for 72 h. After cooling to room temperature, the crystals were isolated by filtration, then washed with DMF, EtOH and dried at 50 °C for 12 h (Yield: 62% based on L2). The crystal data of ZJU-X102 is presented in **Table S3**.

Synthesis of $Sr^{2+}\subset$ZJU-X100. The synthesized ZJU-X100 was immersed in the $SrCl_2$ saturated solution. The solution was replenished more than 5 times for 1 day. The crystal data of $Sr^{2+}\subset$ZJU-X100 is presented in **Table S4**.

Synthesis of $Cs^{+}\subset$ZJU-X102. The synthesized ZJU-X102 was immersed in the CsCl saturated solution. The solution was replenished more than 5 times for 1 day. The crystal data of $Cs^{+}\subset$ZJU-X102 is presented in **Table S5**.

Materials characterization

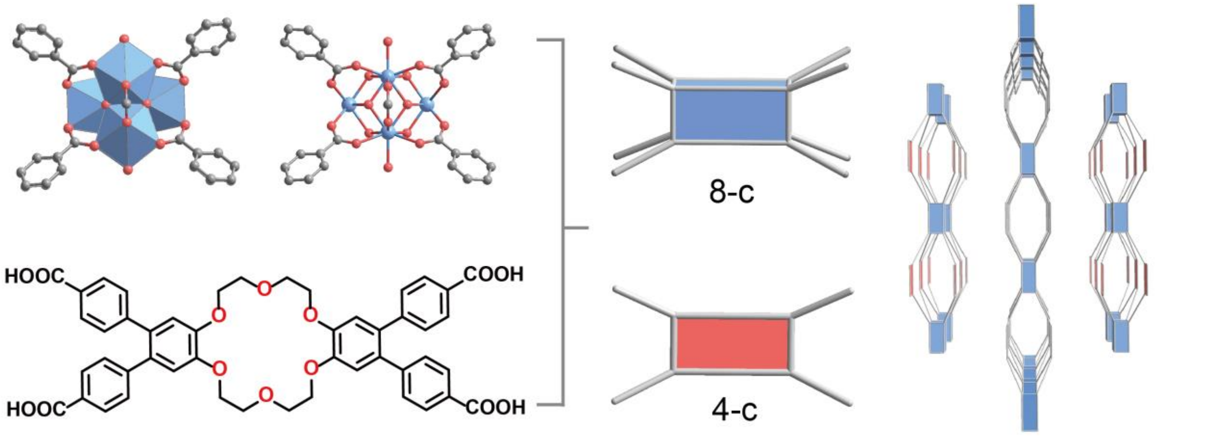

Figure S1. 8-connected Zr nodes, $Zr_6O_4(\mu_3\text{-}OH)_4(\mu_1\text{-}OH)_2(HCOO)_2(H_2O)_2$ and L1 used in ZJU-X100 (L1 = 4,4',5,5'-terabenzoic acid dibenzo-18-crown-6) construct the topology of $\{4^{20}.6^8\}\{4^6\}_2$. Atom colors: Zr, ocean blue; O, red; C, light grey. Hydrogen atoms were omitted for clarity. Atom colors: Zr, ocean blue; O, red; C, light grey. Hydrogen atoms were omitted for clarity.

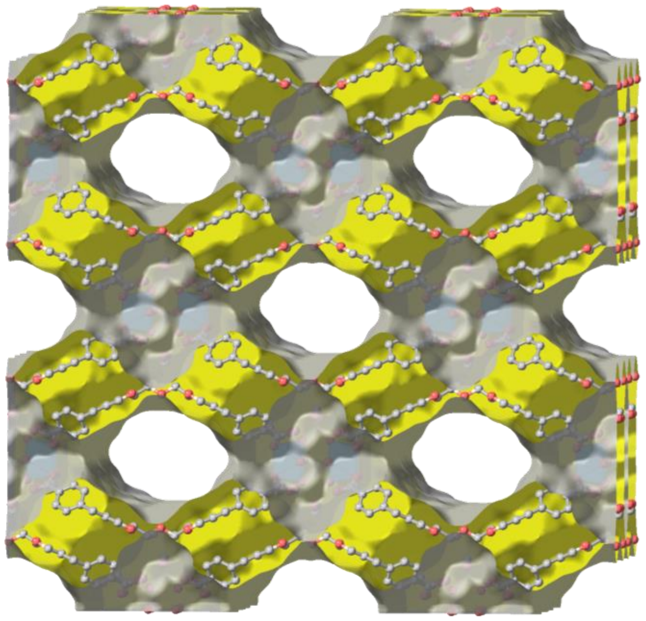

Figure S2. The estimated solvent-accessible void volume of ZJU-X100. Atom colors: Zr, ocean blue; O, red; C, light grey. Hydrogen atoms were omitted for clarity.

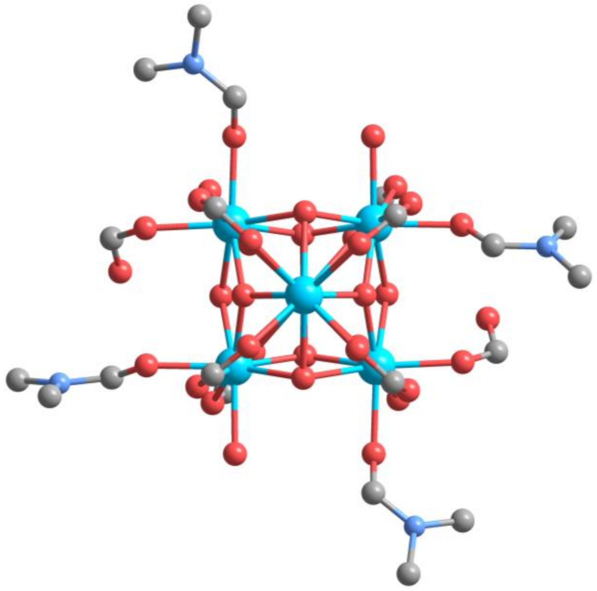

Figure S3. Coordination mode of Zr_6 node in ZJU-X102. Atom colors: Zr, ocean blue; O, red; C, light grey. Hydrogen atoms were omitted for clarity.

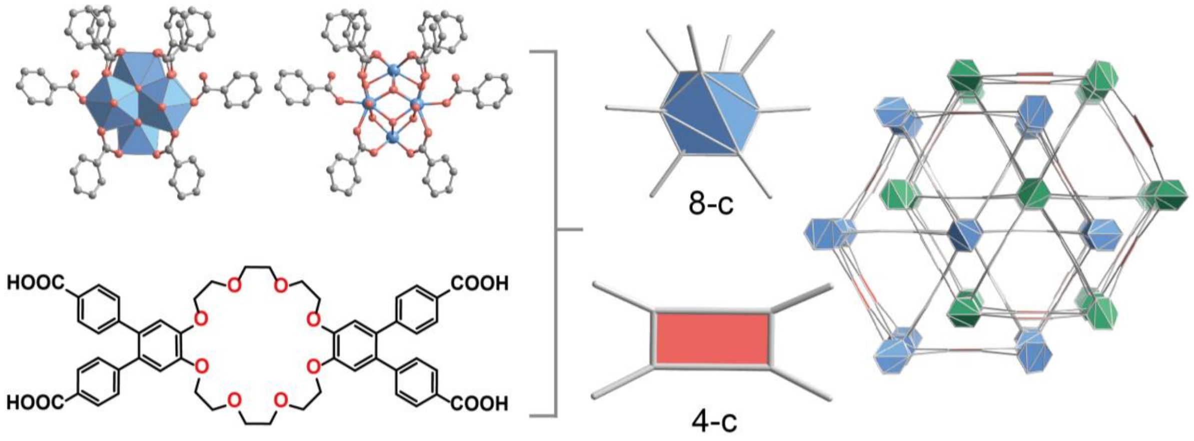

Figure S4. 8-connected Zr nodes, $Zr_6O_4(\mu_3\text{-}OH)_4(\mu_1\text{-}OH)(HCOO)_2(H_2O)$ and L2 used in ZJU-X102 (L2 = 4,4’,5,5’-terabenzoic acid dibenzo-24-crown-8) construct the topology of $\{4^{14}.6^{11}.8^3\}\{4^3.6^3\}_2$. Atom colors: Zr, ocean blue; O, red; C, light grey. Hydrogen atoms were omitted for clarity.

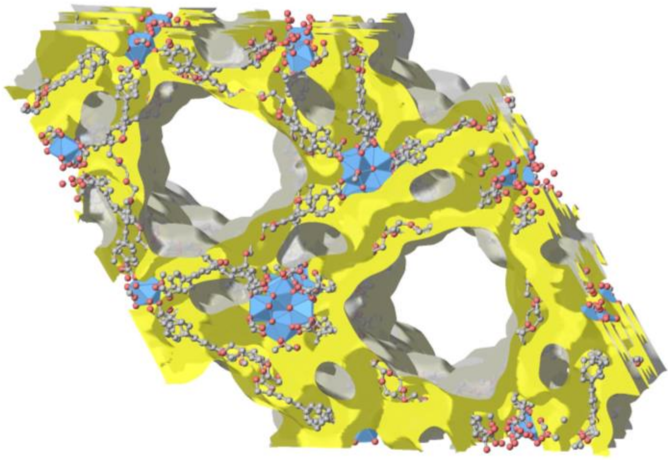

Figure S5. The estimated solvent-accessible void volume of ZJU-X102. Atom colors: Zr, ocean blue; O, red; C, light grey. Hydrogen atoms were omitted for clarity.

Hydrolytic and Solvent Stability Measurements. ZJU-X100 and ZJU-X102 were soaked in NaOH or HCl solution in the pH range from 0 to 11 for 12 h at room temperature. ZJU-X100 were soaked in 12 M HCl solution and solvents with different polarity (MeOH, Acetone, Cyclohexane, DMF, CH_3CN). The samples were collected and dried for powder X-ray diffraction measurements.

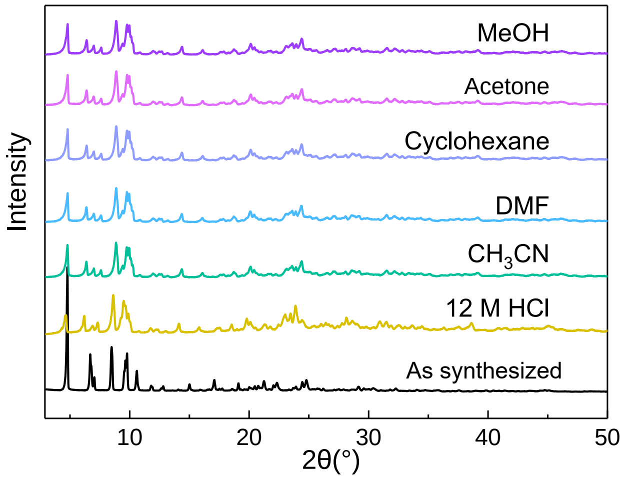

Figure S6. Powder X-ray diffraction patterns of ZJU-100 after exposure in different solution.

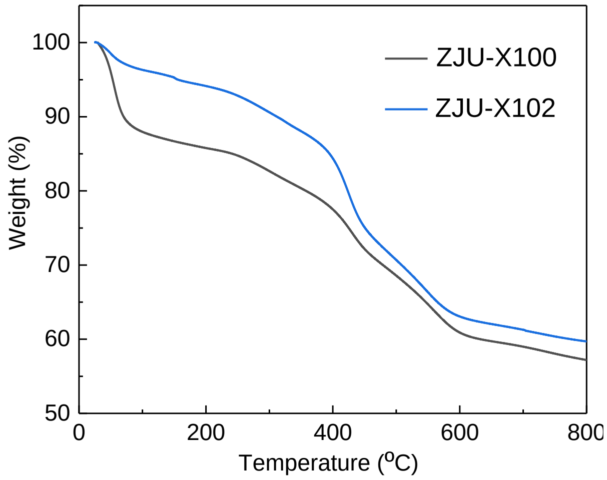

Figure S7. TG curves of ZJU-X100 and ZJU-102.

Sr^{2+} and Cs^{+} extraction experiments

Sorption Kinetics Experiments. The sorption kinetics experiments were carried by dispersing ZJU-X100 and ZJU-X102 into Sr^{2+} and Cs^{+} solution with a solid-liquid ratio of 1:1, respectively. The mixture was stirred at room temperature. The supernatant solution was collected by syringe in different time intervals from 1 to 60 min, then filtered by 0.22 µm nylon membrane filters. The filtrates of residual Sr^{2+} and Cs^{+} were diluted with 5% HNO_3 and the concentrations of Sr^{2+} and Cs^{+} were determined by ICP–MS. The removal percentages were calculated by the equation as follows:

$$R_e = \frac{(C_0 - C_t)}{C_0} \times 100\% \qquad (1)$$

where C_0 (mg/L) is the initial concentration, C_t (mg/L) is the concentration of Sr^{2+} or Cs^{+} after stirring at different times.

Table S1. The adsorption kinetics of Sr^{2+} and Cs^{+} by some typical sorbents compared with ZJU-X100 and ZJU-X102.

Pollutant	Materials	Equilibrium time	Ref

Strontium	KMS-1	> 2 h	[1]
	Go-Hap	2 h	[2]
	Crown@MPPPs	50 min	[3]
	DCH18C6-functionalized resin	1 h	[4]
	FJSM-SnS	60 min	[5]
	FJSM-InMOF	2040 min	[6]
	SNU-200	360 min	[7]
	MOF-18Cr6	60 min	[8]
	SZ-7	5 min	[9]
	**ZJU-X100**	**1 min**	**This work**
Cesium	KMS-1	5 min (65 ℃)	[1]
	KMS-2	10-15 h	[10]
	FJSM-SnS	30 min	[5]
	K-MPS-1	15 min	[11]
	NaFeTiO	200 min	[12]
	Nd-BTC	30 min	[13]
	$[(CH_3)_2NH_2][UO_2(L_2)]\cdot 0.5DMF\cdot 15H_2O$	20 min	[14]
	$[(CH_3)_2NH_2]_4[(UO_2)_4(TBAPy)_3]\cdot 22DMF\cdot 37H_2O$	20 min	[15]
	AMP-PAN	35 min	[16]
	**ZJU-X102**	**1 min**	**This work**

Sorption Isotherm Experiments. The sorption isotherm experiments were measured by exposing ZJU-X100 and ZJU-X102 into different concentrations of Sr^{2+} and Cs^{+} solution with a solid-liquid ratio of 1:1. The samples were stirred for 12 h at room temperature and filtered by 0.22 µm nylon membrane filters. The concentrations of residual Sr^{2+} and Cs^{+} in aqueous solution were determined by ICP–MS. The sorption capacity of ZJU-X100 and ZJU-X102 toward Sr^{2+} or Cs^{+} were calculated by the following equation:

$$q_e = \frac{(C_0 - C_e)}{m} V \qquad (2)$$

Where C_0 (mg/L) is the initial concentration, C_e (mg/L) is the equilibrium concentration, m (g) is the mass of sorbent, and V (L) is the volume of the solution.

Langmuir and Freundlich sorption models were used to fit the sorption isotherms. The Langmuir isotherm model is expressed as given below:

$$\frac{C_e}{q_e} = \frac{1}{q_m K_L} + \frac{C_e}{q_m} \quad (3)$$

where q_e (mg/g) is the sorption capacity at equilibrium, q_m (mg/g) is the maximum sorption capacity and K_L is the Langmuir constant, C_e (mg/L) is the equilibrium concentration of Sr^{2+} and Cs^+. The Freundlich isotherm model is shown as follows:

$$\ln q_e = \ln K_F + \frac{1}{n}\ln C_e \quad (4)$$

where K_F is a constant about sorption capacity and n is related to the intensity of sorption.

Sorption Selectivity Experiments. Selectivity experiments of ZJU-X100 and ZJU-X102 toward Sr^{2+} and Cs^+ were performed by adding ZJU-X100 and ZJU-X102 into 10 ppm of Sr^{2+} and Cs^+ solution containing corresponding proportions of competing anions with a solid-liquid ratio of 1:1, respectively. The solution was stirred for 12 h at room temperature. The samples were filtered and the concentrations of residual Sr^{2+} and Cs^+ were analyzed by ICP–MS, respectively.

Sorption Mechanism

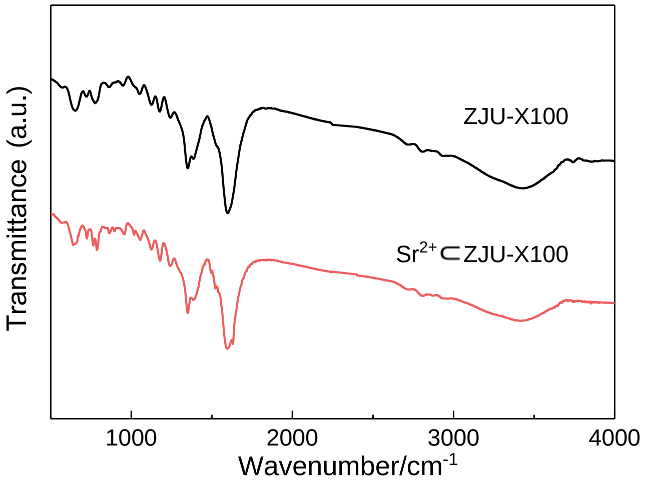

Figure S8. FT-IR spectra of ZJU-100 and Sr^{2+}⊂ZJU-100.

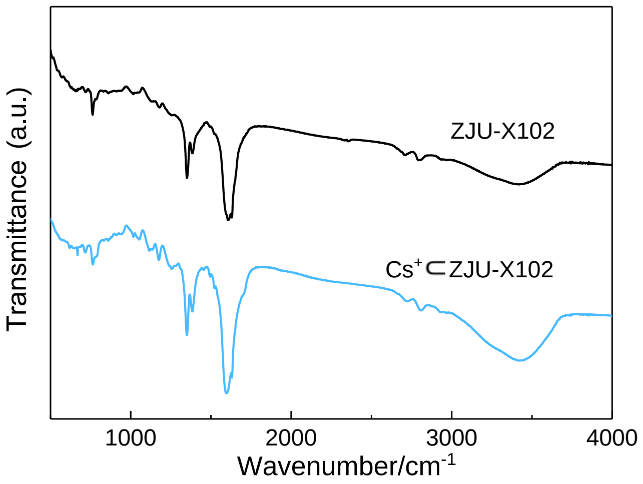

Figure S9. FT-IR spectra of ZJU-102 and Sr^{2+}⊂ZJU-102.

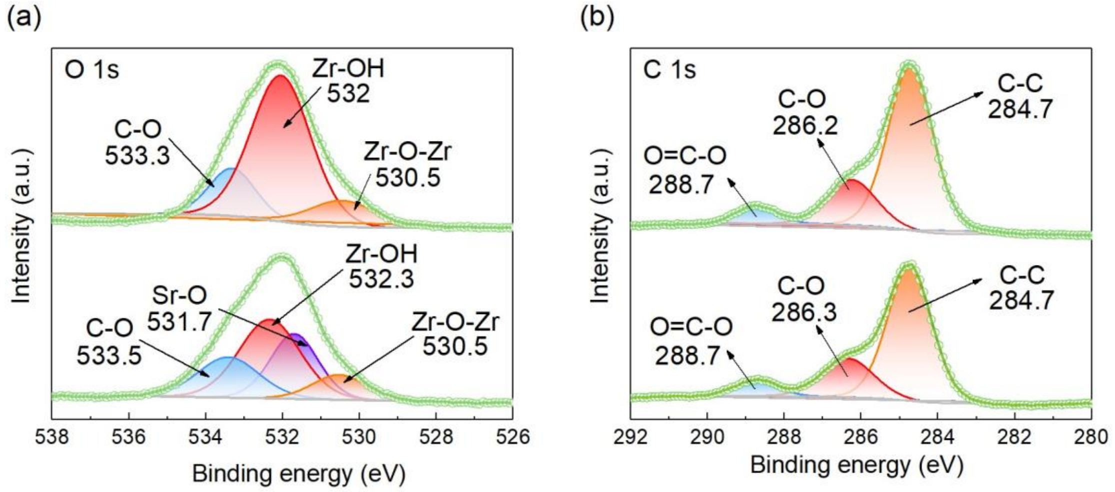

Figure S10. High-resolution a) O 1s and b) C 1s spectra of ZJU-X100 before and after Sr^{2+} adsorption.

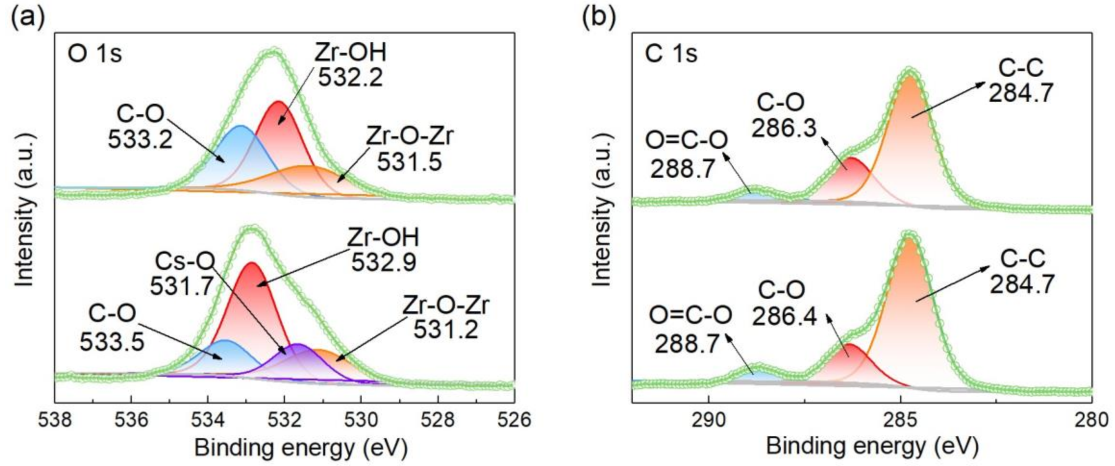

Figure S11. High-resolution a) O 1s and b) C 1s spectra of ZJU-X102 before and after Cs^{+} adsorption.

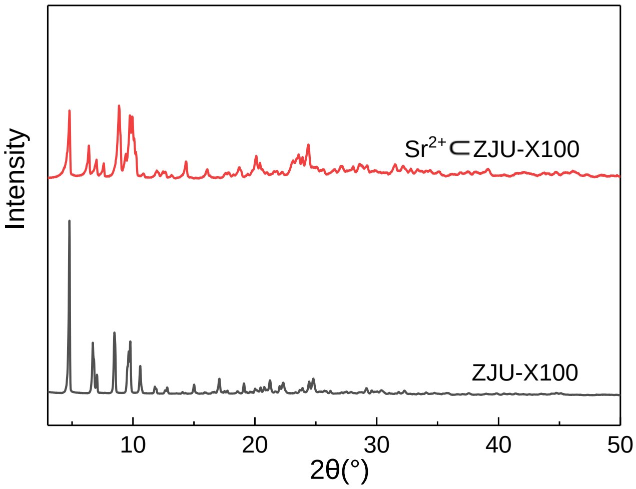

.

Figure S12. Powder X-ray diffraction patterns of ZJU-100 and Sr^{2+}⊂ZJU-100.

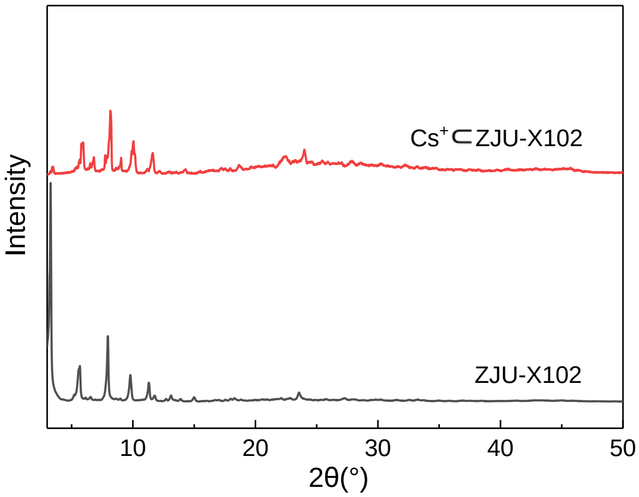

Figure S13. Powder X-ray diffraction patterns of ZJU-102 and Cs^{+}⊂ZJU-102.

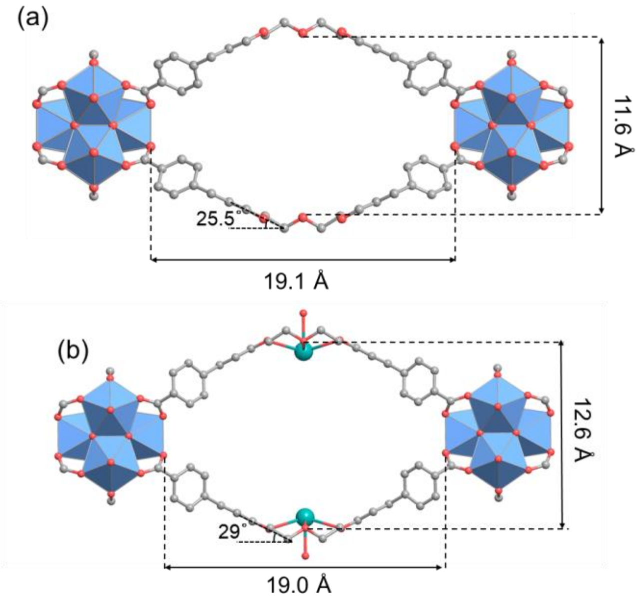

Figure S14. Change of diamond holes of ZJU-X100 a) before and b) after adsorbing Sr^{2+}. Atom colors: Zr, ocean blue; Sr, green; O, red; C, light grey. Hydrogen atoms were omitted for clarity.

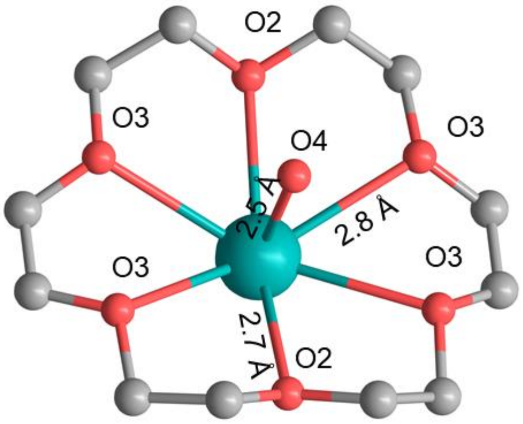

Figure S15. Coordination environment and bond length information of Sr^{2+} in 18-crown-6 group of ZJU-X100. Atom colors: Zr, ocean blue; Sr, green; O, red; C, light grey. Hydrogen atoms were omitted for clarity.

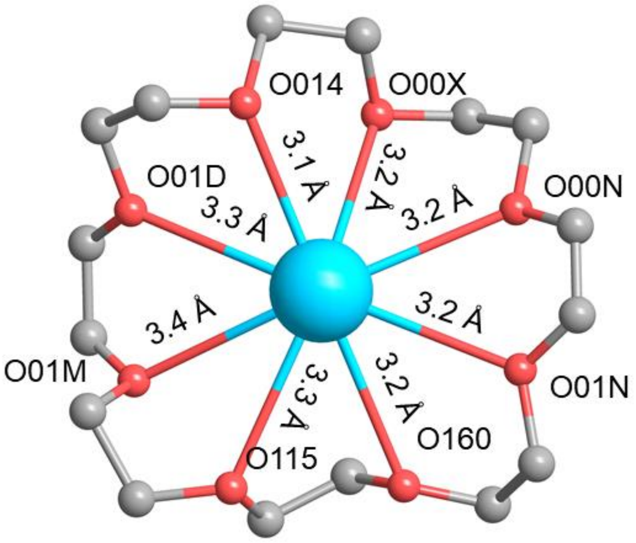

Figure S16. Coordination environment and bond length information of Cs^+ in 24-crown-8 group of ZJU-X102. Atom colors: Zr, ocean blue; Cs, sky blue; O, red; C, light grey. Hydrogen atoms were omitted for clarity.

Table S2. Crystallographic Data and Structure Refinement Parameters of ZJU-X100.

CCDC	2301729
empirical formula	$C_{98}H_{66}O_{44}Zr_6$
temperature/K	200.15
fw/(g/mol)	2428.30
cryst syst	orthorhombic
space group	*Cmmm*
a/Å	26.3044(8)
b/Å	25.7724(12)
c/Å	12.6394(5)
α/deg	90
β/deg	90
γ/deg	90
volume/Å^3	8568.6(6)
Z	2
ρ_{calc}/(g/cm^3)	0.941

μ/mm^{-1}	3.361
reflns collected	55082
indep reflns	4205
GOF on F^2	1.027
R_1 [$I > 2\sigma(I)$]	0.0788
wR_2 [$I > 2\sigma(I)$]	0.2086

Table S3. Crystallographic Data and Structure Refinement Parameters of ZJU-X102.

CCDC	2299622
empirical formula	$C_{122}H_{138}N_6O_{50}Zr_6$
temperature/K	193.00
fw/(g/mol)	3035.70
cryst syst	trigonal
space group	*R* -3*c*
a/Å	54.932(3)
b/Å	54.932(3)
c/Å	37.744(3)
α/deg	90
β/deg	90
γ/deg	120
volume/Å^3	98636(14)
Z	18
ρ_{calc}/(g/cm^3)	0.920
μ/mm^{-1}	1.815
reflns collected	345916
indep reflns	22495
GOF on F^2	1.061
R_1 [$I > 2\sigma(I)$]	0.0745
wR_2 [$I > 2\sigma(I)$]	0.2441

Table S4. Crystallographic Data and Structure Refinement Parameters of Sr^{2+}⊂ZJU-X100.

CCDC	2299617
empirical formula	$C_{97}H_{75.2}O_{46}Sr_2Zr_6$
temperature/K	301.15
fw/(g/mol)	2699.33
cryst syst	orthorhombic
space group	*Cmmm*
a/Å	27.638(6)
b/Å	26.210(3)
c/Å	11.8556(18)
α/deg	90
β/deg	90
γ/deg	90
volume/Å^3	8588(2)
Z	2
ρ_{calc}/(g/cm^3)	1.044
μ/mm^{-1}	4.135
reflns collected	19009
indep reflns	4156
GOF on F^2	1.041
R_1 $[I > 2\sigma(I)]$	0.0981
wR_2 $[I > 2\sigma(I)]$	0.1231

Table S5. Crystallographic Data and Structure Refinement Parameters of Cs^+⊂ZJU-X102.

CCDC	2306608
empirical formula	$C_{228}H_{216}Cs_4N_6O_{98}Zr_{12}$
temperature/K	100.00
fw/(g/mol)	6234.33
cryst syst	trigonal

space group	*R3c*
a/Å	50.9885(5)
b/Å	50.9885(5)
c/Å	37.6460(4)
α/deg	90
β/deg	90
γ/deg	120
volume/Å^3	84760.6(19)
Z	9
ρ_{calc}/(g/cm^3)	1.099
μ/mm^{-1}	6.106
reflns collected	194778
indep reflns	32606
GOF on F^2	1.011
R_1 $[I > 2\sigma(I)]$	0.0768
wR_2 $[I > 2\sigma(I)]$	0.2172

^{1}H NMR Spectra for Compounds

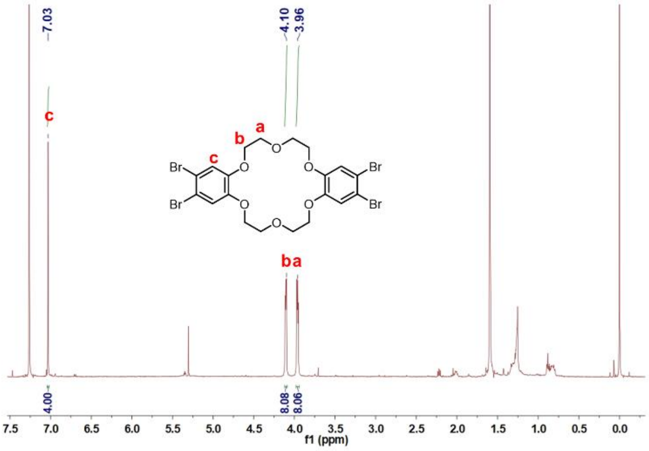

Figure S17. ^{1}H NMR spectrum of 4,4',5,5'-tetrabromodibenzo-18-crown-6. (500 MHz, $CDCl_3$, 298 K)

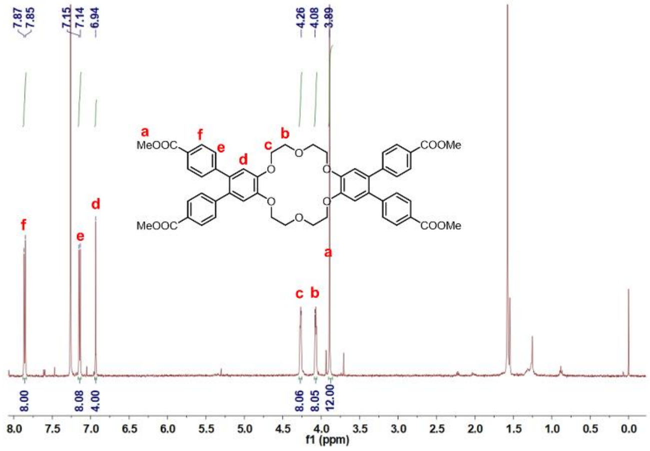

Figure S18. ^{1}H NMR spectrum of 4,4',5,5'-tetrabenzoate dibenzo-18-crown-6. (500 MHz, $CDCl_3$, 298 K)

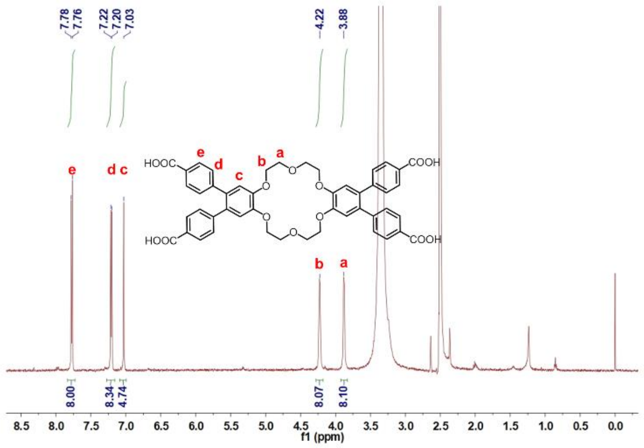

Figure S19. ^{1}H NMR spectrum of 4,4’5,5’-terabenzoic acid dibenzo-18-crown-6. (500 MHz, DMSO-d_6, 298 K)

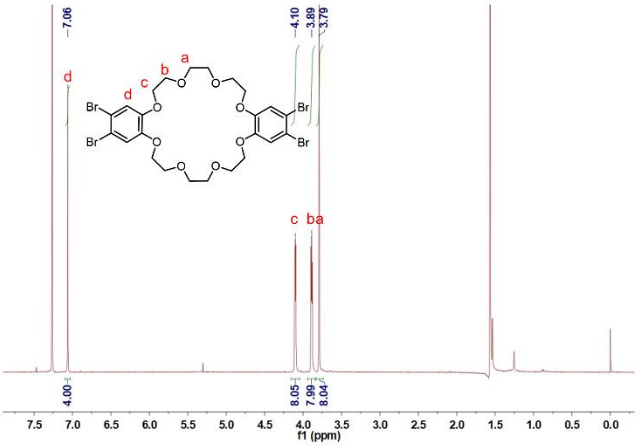

Figure S20. ^{1}H NMR spectrum of 4,4’,5,5’-tetrabromodibenzo-24-crown-8. (500 MHz, $CDCl_3$, 298 K)

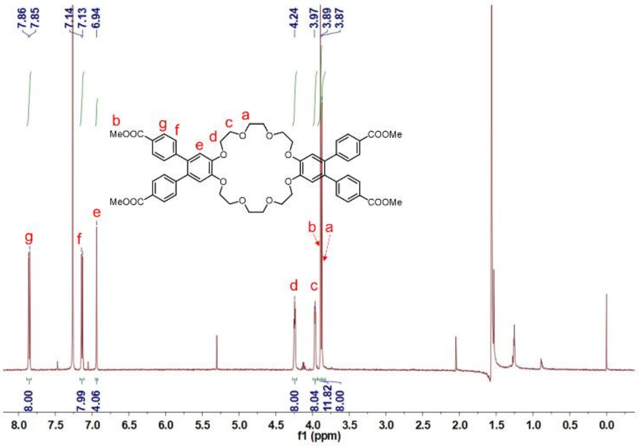

Figure S21. ^{1}H NMR spectrum of 4,4',5,5'-tetrabenzoate dibenzo-24-crown-8. (500 MHz, $CDCl_3$, 298 K)

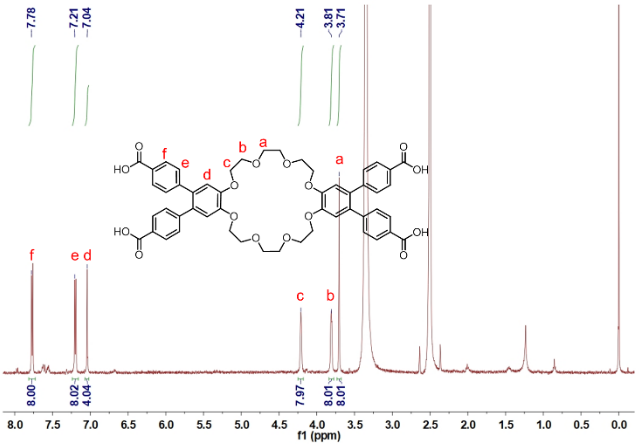

Figure S22. ^{1}H NMR spectrum of 4,4'5,5'-terabenzoic acid dibenzo-24-crown-8. (500 MHz, DMSO-d$_6$, 298 K)

References

[1] M. J. Manos, M. G. Kanatzidis, *J. Am. Chem. Soc.* **2009**, *131* (18), 6599.

[2] T. Wen, X. Wu, M. Liu, Z. Xing, X. Wang, A.-W. Xu, *Dalton transactions* **2014**, *43* (20), 7464.

[3] Y. Song, Y. Du, D. Lv, G. Ye, J. Wang, *J. Hazard. Mater.* **2014**, *274*, 221.

[4] G. Ye, F. Bai, J. Wei, J. Wang, J. Chen, *J. Hazard. Mater.* **2012**, *225*, 8.

[5] X. H. Qi, K. Z. Du, M. L. Feng, J. R. Li, C. F. Du, B. Zhang, X. Y. Huang, *J. Mater. Chem. A* **2015**, *3* (10), 5665.

[6] Y. J. Gao, M. L. Feng, B. Zhang, Z. F. Wu, Y. Song, X. Y. Huang, *J. Mater. Chem. A* **2018**, *6* (9), 3967.

[7] C. Guo, M. Yuan, L. He, L. Cheng, X. Wang, N. Shen, F. Ma, G. Huang, S. Wang, *CrystEngComm* **2021**, *23* (18), 3349.

[8] L. Feng, X. Chen, M. Cao, S. Zhao, H. Wang, D. Chen, Y. Ma, T. Liu, Y. Yuan, N. Wang, *Angew. Chem. Int. Ed.* **2023**, e202312894.

[9] J. Zhang, L. Chen, X. Dai, L. Chen, F. Zhai, W. Yu, S. Guo, L. Yang, L. Chen, Y. Zhang, *Chem. Commun.* **2021**, *57* (68), 8452.

[10] J. L. Mertz, Z. H. Fard, C. D. Malliakas, M. J. Manos, M. G. Kanatzidis, *Chem. Mater.* **2013**, *25* (10), 2116.

[11] E. Rathore, P. Pal, K. Biswas, *Chem. - Eur. J.* **2017**, *23* (46), 11085.

[12] P. Amesh, A. Suneesh, K. Venkatesan, R. U. Maheswari, S. Vijayalakshmi, *Sep. Purif. Technol.* **2020**, *238*, 116393.

[13] P. Asgari, S. Mousavi, H. Aghayan, H. Ghasemi, T. Yousefi, *Microchem. J.* **2019**, *150*, 104188.

[14] Y. Wang, Z. Liu, Y. Li, Z. Bai, W. Liu, Y. Wang, X. Xu, C. Xiao, D. Sheng, J. Diwu, *J. Am. Chem. Soc.* **2015**, *137* (19), 6144.

[15] J. Ai, F. Y. Chen, C. Y. Gao, H. R. Tian, Q. J. Pan, Z. M. Sun, *Inorg. Chem.* **2018**, *57* (8), 4419.

[16] A. Nilchi, R. Saberi, M. Moradi, H. Azizpour, R. Zarghami, *J. Radioanal. Nucl. Chem.* **2012**, *292*, 609.
